# Turning to friends in preference to parents for support in early adolescence: does this contribute to the gender difference in depressive symptoms?

**DOI:** 10.3389/frcha.2023.1150493

**Published:** 2023-05-09

**Authors:** Nicky Wright, Helen Sharp, Jessica Gay, Andrew Pickles, Jonathan Hill

**Affiliations:** ^1^ Department of Psychology, Manchester Metropolitan University, Manchester, United Kingdom; ^2^ Department of Primary Care and Mental Health, University of Liverpool, Liverpool, United Kingdom; ^3^ Department of Psychology, University of Liverpool, Liverpool, United Kingdom; ^4^ Department of Biostatistics & Health Informatics, King’s College, London, United Kingdom; ^5^ School of Psychology and Clinical Language Sciences, University of Reading, Reading, United Kingdom

**Keywords:** depression, adolescence, attachment, social support, parent–adolescent relationship, friendship

## Abstract

**Introduction:**

Based on established evidence of gender differences in friendship patterns, and the vulnerability associated with early reliance on friends, we hypothesized that in 13-year-olds, a preference for turning to friends rather than parents for emotional support contributes to the gender difference in depressive symptoms.

**Methods:**

Using a cross-sectional design, 671 adolescents (53.7% girls; mean age 13.11 ± 0.52 years) in a UK birth cohort [Wirral Child Health and Development Study (WCHADS)] reported turning to their parents and to their friends when distressed [Network of Relationships Inventory (NRI)] and depressed [Short Mood and Feelings Questionnaire (SMFQ)]. Preferentially turning to friends was assessed as turning to friends minus turning to parents for support. Analyses used path analysis using the gsem command in Stata.

**Results:**

Girls had higher depressive symptoms than boys (*p* < .001). Consistent with the hypotheses, girls had higher scores than boys for preferentially turning to friends (*p* < .001). Preferentially turning to friends was associated with higher depressive symptoms (*p* < .001), and this mediated the gender difference in depressive symptoms (*p* < .001). The association between preferentially turning to friends and depressive symptoms was stronger for girls than for boys (*p* = .004).

**Conclusions:**

In young adolescents, preferentially turning to friends over parents when distressed is common, and the association between preferentially turning and depressive symptoms is markedly higher in girls than in boys. This reflects either a gender difference in social vulnerability to depression or a greater impact of depression on the reliance on friends instead of parents in girls. While clarifying the directions of influence requires prospective study, these findings provide the first evidence that the assessment of depression in young adolescents should consider the degree of reliance on friends and parents.

## Introduction

Depressive disorders in adults account for more disability-adjusted life years (DALYs) worldwide than any other psychiatric disorder (1 DALY represents the loss of the equivalent of 1 year of full health) (1). The onset of depression is typically in adolescence, with a marked increase over the period of 11–14 years, which is much greater in girls than in boys (2–4). This gender^1^ difference appears to be increasing over time. In a recent publication from a nationally representative survey of adolescents aged 12–17 years in the United States (*n* = 167,783), Daly (5) rates of depression were reported to be 23.4% in girls and 8.4% in boys, and the gender difference had increased from 6.4% to 14.8% between 2009 and 2019. Understanding this gender difference and identifying potential targets for intervention to reduce depression in adolescent girls is a key goal in reducing the lifetime burden associated with depressive disorders.

The gender difference becomes evident in early adolescence when girls’ levels increase much more than boys’. The reasons are complex and include hormonal (6), psychological (7), and social contributions. Based on the available evidence, it is possible that among the social contributions, a vulnerability may arise from the tendency for girls to rely more on friends for emotional support than on their parents. Longitudinal studies suggest that girls show a greater decrease in support from parents and an increase in support from friends in early adolescence (8–10). Findings from a meta-analysis and a systematic review suggest that parental social support is consistently associated with reduced depressive symptoms in adolescence, whereas friendship support shows a small association that is less consistent across studies (11, 12). In addition, in girls more than in boys, a lack of parental support is associated with increased depressive symptoms in childhood and adolescence (11). It is possible that turning more to friends for support in early adolescence could create vulnerability to depression, either as a reflection of a lack of support from parents or because relying on peers for support is risky because they are not yet able to provide adequate support due to their youth. In this study, we investigate the relationship between support seeking from parents and from friends and depression in early adolescence, specifically whether a preferentially turning to friends over parents is associated with a higher risk for depression. We also test whether differences in support seeking to explain the gender difference in depressive symptoms. We define “support seeking” as turning to parents or friends for comfort and support when distressed. Existing evidence is limited by the frequent use of non-validated measures of support seeking (11, 12); in this study, we use an established measure.

Bowlby's (13) attachment theory proposes that infants form an attachment bond with a specific caregiver, with whom they seek proximity and contact, particularly when they are feeling distressed. Bowlby proposed that there is a normative developmental progression from seeking support from parents in infancy and childhood to fulfilling attachment needs with romantic partners in adulthood. This process of transfer of attachment from parents to peers is proposed to begin in adolescence (13–15). Between childhood and adolescence, the attachment system is thought to reorganize from a single attachment to a parent to an attachment hierarchy with multiple figures, including peers (15). Peers become increasingly important during adolescence. Adolescents spend greater amounts of time with their peers (16) and are more concerned with peer acceptance and evaluation. Concerns about these topics, and sexuality, are more likely to be discussed with peers than with parents (17). The increased desire for autonomy and independence during adolescence also encourages adolescents to turn to their peers for support and guidance (15). In addition, advances in cognitive development during adolescence support the use of different figures for different attachment needs (18). Peer attachment relationships are more reciprocal and mutually supportive than parent–child attachments (19). The nature of relationships with parents also changes during adolescence, becoming more egalitarian, which is associated with changes in conflict and closeness between adolescents and parents (8). Thus, overall, the evidence supports Bowlby's (13) hypothesis that there is a developmental progression from parents as attachment figures to adolescents and young adults (14, 20, 21).

However, the attachment construct is multifaceted, and the developmental progression may not be the same for each different element: the secure base effect and another aspect such as comfort-seeking when distressed (14, 21). Much of the conceptualization and measurement of attachment security has focused on emotion regulation with the help of a close other (22). This is the key dimension in assigning attachment security in the Strange Situation Procedure (SSP) (23). A secure child turns to a caregiver for comfort and reassurance when reunited after separation. Insecure attachments are characterised by different variations of less efficient use of a caregiver to calm distress. Although adolescent measures do not assess emotion regulation in interaction with a caregiver, it has been proposed that attachment security assessed, for example, as an attachment interview, reflects interpersonal emotion regulation strategies (24). Interpersonal emotion regulation is also likely to be key to vulnerability to depression. Depression is associated with difficulties in emotion regulation, and several studies have shown associations between attachment status, emotion dysregulation, and depressive symptoms in adolescents (25). There are therefore strong reasons to focus on the adolescent equivalent of turning to parents when distressed, as found in the SSP.

Given that turning to another person when distressed implies a degree of trust and reliance on that person and a need for them to be sufficiently skilled to provide a soothing response, it may be that too early a reliance on friends could create vulnerability. Kobak et al. (15) argued that this would be the case according to their premature reorganization of the attachment hypothesis. According to their theory, if the attachment hierarchy is prematurely reorganized so that peers are used as more important sources of support than parents, there will be a risk for mental health problems. This hypothesis has received some support from two studies that examined the position of friends and parents in attachment hierarchies, as defined by the ranking of attachment figures on “attachment bonding” (defined as closeness, separation distress, and comfort in an emergency). Rosenthal and Kobak (26) found associations between higher peer placement and self-reported internalizing and externalizing problems in adolescents aged 15–18 years. In a study of adolescents aged 11–18 years, Umemura et al. (27) found that higher placement of mothers, fathers, and other family members in the hierarchy was associated with lower teacher-reported externalizing problems and internalizing problems. However, the associations were small and not consistent across the teacher, mother, and self-report measures. Both studies used opportunity samples from middle-income (26) or upper middle-income (27) backgrounds, which limits the generalisability of the findings to general population samples. Both studies were also cross-sectional and therefore cannot inform on the direction of the association between attachment hierarchy placement and mental health symptoms.

Both Rosenthal and Kobak (26) and Umemura et al. (27) are relevant to the current research question; however, neither provided the important contrast between turning to friends vs. turning to parents. Furthermore, neither examined the possibility, considered earlier, that girls may show this preferentially turning to friends more than boys, and thus this may mediate the gender difference in adolescent depression. Neither they nor any other published study tested whether preferentially turning to friends might be more strongly associated with depression in girls than in boys. Such moderator effects have been found elsewhere in the literature. For example, social comparison and feedback-seeking on social media (NESI) and friendship stress (28) are associated with depressive symptoms in adolescents but are moderated by gender, such that the association is particularly strong for girls.

The direction of the association between support and depression is commonly assumed to be that less support confers risk. However, it is also possible that depressed adolescents seek less support from their parents and more support from their friends. Evidence supports a reciprocal relationship between parental support and adolescent depressive symptoms (29–31). In one study, both directional pathways were for girls (30), and in another, the pathways from support to depressive symptoms were stronger for girls (31). There is also some evidence of reciprocal relationships for positive friendship qualities and depressive symptoms with no gender difference (32). Depressed adolescents are also more likely to select and keep friends with similar levels of depression to themselves (33), and evidence suggests that depression levels in friendships become more similar over time (34), with one study finding this only in girls (35). There is evidence that higher-quality friendships are characterized by higher co-rumination, which in turn is associated with increased depressive symptoms (36–38). There is also evidence for differences in these associations with a reciprocal relationship in girls, where co-rumination increases friendship quality, which in turn increases depression, whereas in boys, when co-rumination increased friendship quality, friendship quality itself was not associated with increased depression (37). In another study, co-rumination was found to mediate the association between gender and depressive symptoms in female college students but not in male college students (39).

In light of this discussion, we test five hypotheses in this study. The first two aim to provide evidence in support of well-established findings, and the remainder are novel:

(1) There will be a gender difference in support seeking from parents, with girls showing higher levels of support seeking and increasingly turning to their parents, which is more strongly associated with reduced depressive symptoms in girls than in boys.
(2) There will be a gender difference in support seeking from friends, with girls showing higher levels of support. Based on existing evidence, we do not hypothesize a directional association between support seeking from friends and depressive symptoms, nor do we hypothesize a gender difference.
(3) Preferentially turning to friends, assessed as turning to friends minus turning to parents for support, will be higher in girls than in boys.
(4) Preferentially turning to friends will be associated with higher depressive symptoms and will mediate the gender difference in adolescent depressive symptoms.
(5) The association between preferentially turning to friends and higher depressive symptoms will be moderated by the child’s gender, so that the association will be substantially stronger in girls than in boys.

## Methods

### Sample size

The study is embedded in the Wirral Child Health and Development Study (WCHADS), a prospective epidemiological study of child development in a sample of first-time mothers (*n* = 1,233) (40). The socioeconomic conditions in Wirral vary between the deprived inner city and affluent suburbs, with lower numbers from ethnic minorities. The mean age at recruitment in pregnancy was 26.8 ± 5.8 years (range 18–51 years), 41.8% of the sample lived in the most deprived quintile of UK neighborhoods [2003 Indices of Multiple Deprivation (IMD)] (41) and 96.1% were White British. The study collected data in 13 waves from 20 weeks of gestation up to 13 years of age.

The sample analyzed here consists of the adolescents who provided data on all measures included in this report at the age 13 data collection wave (*n* = 671). The mean age of the sample is 13.11 ± 5.2 years, with slightly more girls (*n* = 360, 53.7%) responding than boys (*n* = 311, 46.3%). The majority of adolescents lived in married (*n* = 400, 59.6%) or cohabiting (*n* = 119, 17.7%) parental households, with 6.3% (*n* = 42) living with a parent who had a partner who lived elsewhere and 15.8% (*n* = 106) living with a parent who was either single, divorced, separated, or widowed. A similar proportion of this sample to the originally recruited sample was living in the most deprived quintile of the UK neighborhood at conception (*n* = 362, 39.2%).

### Ethical considerations

Ethical approval for the study was granted by the Cheshire North and West Research Ethics Committee on 27 June 2006 (reference no. 05/Q1506/107) and on 22 December 2014 and 8 June 2020 (reference no. 14/NW/1484). All women gave written informed consent at recruitment and subsequent assessment waves. Child assent was gained at age 13.

## Measures

### Support seeking from parents and friends

The adolescent report of the Network of Relationships Inventory (NRI): Behavioral Systems Version (42) was used to assess the extent to which children sought support from their parents and their friends when distressed. The NRI assesses 12 provisions of close relationships, including five components of social support that relate to caregiving, affiliation, and attachment. For the current study, we focused on only one of these components, “participant seeks a safe haven.” The scale comprises three items: “How much do you seek out this person when you are upset?” “How much do you turn to this person for comfort and support when you are troubled about something?” and “How much do you turn to this person when you are worried about something?” Adolescents were directed to answer the same three questions about the parent with whom they spent the most time and the friend with whom they spent the most time. Items were rated on a 4-point Likert scale ranging from little/none (1) to extremely much (4) and summed to create a total score. A preferentially turning to friend variable was generated by subtracting the total score for parent support from the total score for friend support. The internal consistency of the scales was good: Cronbach's alpha = .88 for parent support and .94 for friend support.

### Depressive symptoms

The adolescent report on the Short Mood and Feelings Questionnaire (SMFQ) (43) was used to assess depressive symptoms. The scale includes 13 items assessing DSM-IV symptoms of depression over the prior 2 weeks. Items are rated on a 3-point scale from 1 = not true to 3 = true.

### Child gender

We use the child’s sex recorded at birth (coded 1 = male and 2 = female) to index gender. Gender has not been systemically assessed in the WCHADS at birth. The study did not complete any face-to-face assessments since age 9, which would have allowed us to sensitively ask about gender identity. In this paper, we refer to gender differences because this is what most studies of social processes in adolescence aim to understand, but we are aware that in a small number of instances in our study, perhaps 1%, a child’s gender is different from their sex.

### Confounding variables

Socioeconomic status was determined using the revised English IMD (41), based on data collected from the UK 2001 census. According to this system, postcode areas in England are ranked from most deprived (i.e., an IMD of 1) to least deprived (i.e., an IMD of 32,482) based on neighborhood deprivation in seven domains: income, employment, health, education and training, barriers to housing and services, living environment, and crime. All mothers were given an IMD rank according to the postcode of the area in which they lived during pregnancy and were assigned to a quintile based on the UK distribution of deprivation (1 = most deprived, 5 = least deprived). A binary variable reflecting 1 = living in the most deprived quintile vs. 0 = quintiles 2–5 was used in the analysis. The age in months at which the questionnaire was completed was calculated using the date of completion and date of birth. Pubertal status was assessed using the adolescent report of the Pubertal Development Scale (PDS) (44). The PDS is a self-report measure for adolescents that assesses the development of growth in height, body hair, and skin changes (three items), plus two gender-specific items (voice deepening and growth of facial hair in boys and the growth of breasts and menstruation in girls). Each item, except for menarche, which is rated 1 = no or 4 = yes, is rated on a 4-point scale (from has not yet begun = 1 to seems complete = 4). The items were converted into five maturation categories (ranging from pre-pubertal to post-pubertal) to reflect the Tanner stages according to Carskadon and Acebo (45) This involves summing the scores for boys and girls separately and assigning them to the maturation categories according to the total score and a set of rules laid out in Carskadon and Acebo (45) (e.g., to be classified as late pubertal, a girl must have experienced menarche and have a total score <7). For this study, maturation stages 1 and 2 were grouped to represent “early pubertal” (*n* = 94, 30.2% for boys, and *n* = 13, 3.6% for girls), development category 3 represents “mid-pubertal” development (*n* = 133, 44.4% for boys, and *n* = 76, 21.1% for girls), and categories 4 and 5 were grouped to represent “late pubertal development” (*n* = 79, 25.4% for boys, and *n* = 271, 75.3% for girls) (46). Dummy variables reflecting early- and mid-pubertal development were included as confounding variables, with late pubertal development serving as the reference.

### Analysis plan

Bivariate associations were examined using Spearman's correlations. All analyses were conducted in Stata version 17 (47). Adolescent depression scores were highly skewed, with a mode of 0, and therefore not suitable for transformation. Therefore, the gsem command in Stata was used to test the main study hypotheses, using path analysis with depression scores modeled with a negative binomial distribution. Indirect effects were tested using the nlcom command. Interactions were plotted using the margins command in Stata, showing the association between support-seeking and adolescent outcomes in boys and girls. Interactions were explored using the margins command to estimate the marginal effects of support seeking at 1 SD above or below the mean and at the mean on depressive symptoms in boys and girls separately. Variables were standardized before generating interaction terms.

## Results

Descriptive statistics for support-seeking and depressive symptoms for the total sample (*n* = 671) and by gender are shown in Table 1. Due to significant skewness, a Kolmogorov–Smirnov test was used to check for a gender difference in depressive symptoms, which was highly significant (*p* < .001), with girls showing higher mean depression scores (Cohen's *d* = .35).

**Table 1:** Descriptive statistics for the key study variables in boys and girls.

	Total sample		Boys *n* = 311		Girls *n* = 360	
	Mean	SD	Mean	SD	Mean	SD
Depression	5.79	5.85	3.99	4.38	7.35	6.48
Turning to parents	7.60	2.58	7.51	2.40	7.67	2.73
Turning to friends	6.64	2.74	5.33	2.25	7.77	2.63
Preferential turning to friends	−.96	3.39	−2.18	2.78	.10	3.51

Bivariate associations between hypotheses and confounding variables are shown in Table 2 for boys and girls separately. Parental support seeking was significantly negatively associated with symptoms of depression, strongly in girls and less so in boys. Friend support seeking was very weakly associated with symptoms of depression in boys but not in girls. Friend and parental support were positively associated in both genders, but more strongly in boys. Preferential friend support seeking was strongly associated with symptoms of depression in girls and not at all in boys. Among the confounding variables, age and increased pubertal development were weakly associated with increased depression symptoms in girls, and increased pubertal development was associated with less parental support seeking.

**Table 2:** Spearman's correlations between the study variables in boys and girls, with boys shown on the top diagonal and girls on the bottom diagonal.

	Depression	Parents	Friends	Preferential friend	Age	IMD	Puberty
Depression		−.19**	−.12*	.05	−.06	.11	.01
Parental support	−.51***		.30***	−.64***	−.05	.05	−.06
Friend support	.02	.15**		.49***	−.01	.01	−.01
Preferential friend	.41*	−.66***	.60		.05	−.04	.05
Age at assessment	.13*	−.05	−.06	−.03		.14*	.37***
IMD most deprived	.01	.01	−.01	−.01	−.01		.10
Increased pubertal development	.13*	−.15**	−.08	.06	.30***	.06	

IMD most deprived, indices of multiple deprivation most deprived quintile.

* *p* < .05, **p < .010, ***p < .001.

### Turning to parents

**Figure 1:**
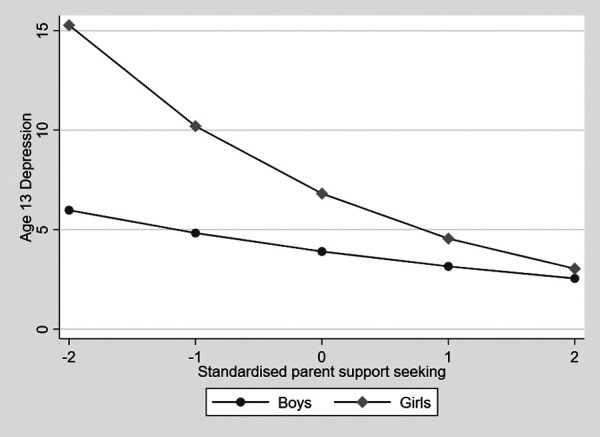
The association between seeking support from parents and adolescent depressive symptoms in boys and girls.

In a test of Hypothesis 1, in the one-way analysis of variance (ANOVA) accounting for confounders, there was a significant gender difference in turning to parents for support (score based on a Likert scale of 1–7 ), with girls showing higher levels, although this was a small effect (eta squared = .01, 95% CI 0 to –.03, *p* = .044). In the gsem model, there was a negative association, with decreased turning to parents for support associated with increased depression symptoms (estimate –.35, 95 CI –.41 to –.25, *p* < .001). Means and standard deviations are shown in Table 1. Given the significant gender difference in turning to parents, the mediation of the gender difference on depression symptoms was tested by using the nlcom command in Stata to estimate the indirect effect of gender on depression via turning to friends and the direct effect of gender on depression symptoms. The nlcom command uses the delta method to calculate the standard error. The indirect effect of gender via turning to parents on depression symptoms was not significant (indirect effect estimate = –.06, 95% CI –.12 to .01, *p* = .075; direct effect estimate = .48, 95% CI .28 to .67, *p* < .001) indicating no mediation. As predicted, the negative association between turning to parents and depression symptoms was significantly stronger for girls than for boys (interaction term estimate = –.20, 95% CI –.35 to –.04, *p* = .012). This is shown in Figure 1, where it can be seen that decreasing support seeking from parents is associated with increased depression symptoms in girls but not in boys. This was explored by estimating the marginal effects of turning to parents on depression symptoms at 1 SD above and below the mean and the mean in girls and boys separately. The marginal effect of turning to parents was highest for girls 1 SD below the mean (marginal effect = 10.24, SE .23) and progressively lower for those at the mean (marginal effect = 6.90, SE .14) and then 1 SD above the mean (marginal effect = 4.65, SE .15). Boys also showed the largest effect at 1 SD below the mean, which decreased from the mean to 1 SD below, but the differences were smaller (marginal effect = 4.72, SE .18, marginal effect = 3.88, SE .11, and marginal effect = 3.18, SE .15, respectively).

### Turning to friends

**Figure 2:**
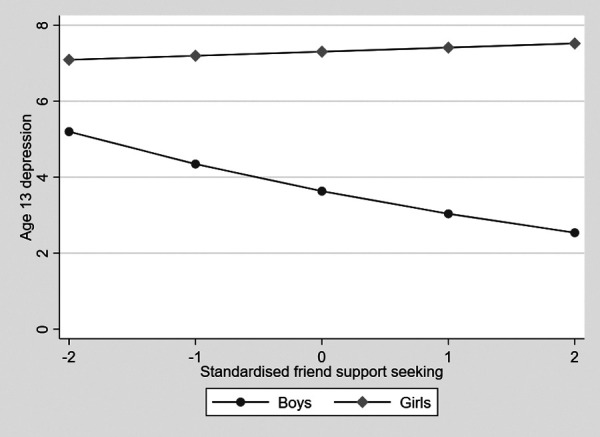
The association between seeking support from friends and symptoms of adolescent depression in boys and girls.

To test Hypothesis 2, with confounders, there was a significant gender difference in turning to friends for support in a one-way ANOVA accounting for, with girls showing higher levels and a moderate to large effect size (partial eta squared = .16, 95% CI .11 to .21, *p* < .001). Means and standard deviations are shown in Table 1. In the gsem model, turning to friends for support was not associated with depression symptoms (estimate = –.02, 95% CI –.05 to .01, *p* = .237). Because there was no gender difference in turning to friends, mediation was not tested. There was a significant interaction between child gender and turning to friends in predicting depression symptoms (estimate = –.17, 95% CI –.32 to –.03, *p* = .018). This is shown in Figure 2, where it can be seen that decreasing support seeking from friends is associated with increased depression symptoms in boys but not in girls. This was examined by estimating the marginal effects of turning to parents on depression symptoms at 1 SD above and below the mean and the mean for boys and girls separately. The marginal effect was highest for boys 1 SD below the mean for turning to friends (marginal effect = 4.56, SE .16) and progressively lower for those at the mean (marginal effect = 3.81, SE .14), and then 1 SD above (marginal effect = 3.18, SE .21). For girls, there was no difference between the three levels (marginal effect = 6.92, SE.25, marginal effect = 7.02, SE.16, and marginal effect = 7.13, SE .17, respectively).

**Figure 3:**
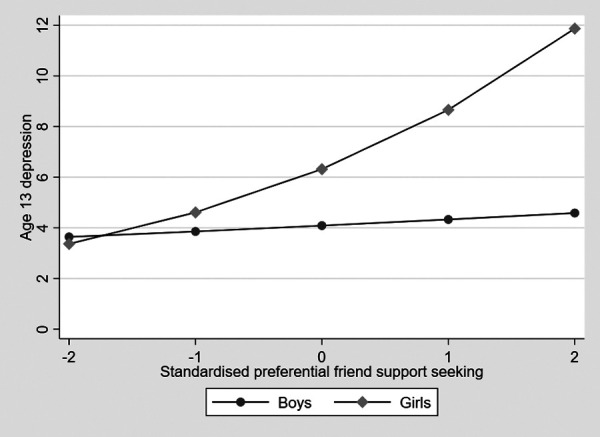
The association between preferentially turning to friends and adolescent depressive symptoms in boys and girls.

### Preferentially turning to friends

Hypothesis 3 was supported in the one-way ANOVA accounting for confounders, where there was a significant gender difference in preferentially turning to friends for support, with girls showing higher levels with a moderate to large effect size (partial eta squared = .07, 95% CI .03 to .11, *p* < .001). In the gsem model, preferentially turning to friends for support was associated with increased depression symptoms (estimate = .22, 95% CI .14 to .30, *p* < .001). Given this finding, we examined the mediation of gender differences in depression symptoms by preferentially turning to friends (Hypothesis 4). There was a significant indirect effect of adolescent gender on depression symptoms via preferentially turning to friends (indirect effect estimate = .13, 95% CI .09 to .21, *p* < .001; total effect estimate = .48, 95% CI .28 to .69, *p* < .001). In a test of Hypothesis 5, the association between preferentially turning to friends and depression symptoms was significantly greater for girls than for boys (interaction term estimate = .26, 95% CI .09 to .42, *p* = .002). This is shown in Figure 3, where it can be seen that increasing preferentially turning to friends is associated with increased depression symptoms in girls but not in boys. This was examined by estimating the marginal effects of preferentially turning to friends on depression symptoms at 1 SD above and below the mean and at the mean for girls and boys separately. The marginal effect was largest for girls 1 SD above the mean for turning to parents (marginal effect = 8.41, SE .56) and decreased progressively for those at the mean (marginal effect = 6.14, SE .36) and then 1 SD below (marginal effect = 4.48, SE .39). Boys also showed the greatest effect at 1 SD above the mean, which decreased from the mean to 1 SD below, but the differences were small (marginal effect = 3.99, SE .32; marginal effect = 4.23, SE .30; and marginal effect = 3.99, SE .32, respectively).

## Discussion

In this study, we examined the associations based on hypotheses about the role of friends and parents in providing emotional support to 13-year-olds. Based on the idea that reliance on friends at this age may create vulnerability, we hypothesized that a higher preferentially turning to friends score (turning to friends minus turning to parents) would be associated with higher depressive symptoms. This hypothesis was supported. Given the well-established difference in the levels of depressive symptoms between boys and girls at this age, we then considered the possibility that the vulnerability associated with preferentially turning to friends was implicated in this gender difference. Before examining mediation, we asked whether levels of preferentially turning to friends were higher in girls than in boys, and found a large difference. In mediation analyses for the gender difference in depressive symptoms, we found an indirect effect of preferentially turning to friends. We also considered the possibility that the vulnerability to depression associated with preferentially turning to friends might be greater in girls than in boys. Consistent with this hypothesis, we found that child gender moderated the association between preferentially turning to parents and depressive symptoms. While the findings are reported in the context of possible mechanisms for adolescent vulnerability to depression, in this cross-sectional study, it is not possible to tell which of the associations resulted from the effects of low mood on relationships with friends and parents.

The study was conducted in the context of Bowlby's (13) hypothesis that there is a normative developmental transition from fulfilling attachment needs with parents to peers, and Kobak's proposal (15) that if this occurs too early, it creates vulnerability because peers are not yet ready to provide the necessary emotional resources. The findings are consistent with studies described earlier in this paper showing that assigning friends a higher position in the attachment hierarchy across different kinds of relationships is associated with higher mental health problems (26, 27). Our findings also replicated the well-established gender difference in depressive symptoms among young adolescents(2–4). To our knowledge, previous studies have not asked whether a premature reliance on friends might help explain this difference. More generally, there is little evidence on the possible role of social support in explaining the greater vulnerability of adolescent girls to depression. To our knowledge, no previous study has examined social support. There is some evidence implicating social processes, such as greater friend- and peer-related stress and greater co-rumination (39), in explaining the gender difference. The findings reported here, consistent with both mediation and moderation by preferentially turning to friends because of the gender difference in depression, are therefore both novel.

We also replicated some other well-established findings in the literature in this sample. These include a gender difference in seeking support from friends, with girls showing greater support seeking (8, 21, 48). Parental support was significantly associated with reduced depressive symptoms, and this was stronger for girls (11). We also found no association between friend support seeking and depressive symptoms, which is consistent with previous meta-analyses that have found small and inconsistent associations (11, 12). Our finding of a strong positive association between preferentially turning to friends and depressive symptoms is consistent with the hypothesis that reliance on friends to perform functions typically performed by parents in early adolescence may create psychological vulnerability. A negative association between turning to friends and depressive symptoms might have been expected based on the idea that friends are a valuable source of support when this is complemented by parental support. This finding may, however, indicate that while friends are an important source of support in a broader sense, they are not an important source of comfort in times of distress. However, in the moderation analysis, turning to friends was associated with depressive symptoms in boys, consistent with a risk effect where lower turning to friends was associated with higher depressive symptoms. This was not expected, and gender differences in the association between friend support and depressive symptoms have not been found in previous meta-analyses (11, 12).

## Strengths and limitations

A major strength of the study is that the data were generated from a large, well-characterized general population birth cohort. The recruitment method limited systematic bias in the sample by approaching every first-time pregnant woman attending an antenatal clinic over a defined period of time. This was the only UK NHS facility within the well-defined geographical area of the Wirral. The deprivation profile of the study sample closely matched the published information on the profile of the region. The analyses controlled for pubertal status, which has been associated with reduced attachment to parents in girls (49) and increased depressive symptoms in girls (6). We focused on a sample with a narrow age range in early adolescence, the time when the gender difference in depression emerges.

Nevertheless, generalization to the UK population may be limited in two ways, both related to the demographic profile of the Wirral. First, the rates of deprivation are higher than in the UK as a whole, so it is not possible to rule out that the patterns of association are a function of area deprivation. Second, there are very few ethnic minority families in Wirral, and this was reflected in the study sample. Further studies with a sufficient representation of one or more ethnic minority groups are needed to test whether the findings are moderated by ethnicity.

An important limitation of the current study is its cross-sectional design. It is therefore possible that the associations that we report are due to a greater impact of depression on preferentially turning to friends in girls than in boys. It is also possible that there was a third variable effect that was also gender-dependent. Furthermore, all of the measures used in the study were based on adolescent self-report, which may have inflated the associations through common method variance, and perceptions of turning to friends and parents may have been influenced by low mood. However, the main hypotheses concerned differences between boys and girls, for which common method variance and mood effects would only be relevant if they also varied by child gender, which seems unlikely. The measures were broad and did not make distinctions that might be highly informative, such as whether youth reported turning to same-sex or opposite-sex friends. Similarly, the measure did not differentiate between turning to mothers or fathers.

## Implications of the findings

Regardless of the direction of effects, the findings point to important differences in the mechanisms associated with male and female depression in early adolescence. Given that adolescent depression commonly recurs later in adolescence and into adulthood, these mechanisms may have long-term implications. A priority, therefore, is to use a prospective design to determine the temporal associations between emotional support from friends and parents and depression. This should be examined from childhood through late adolescence or early adulthood.

While there is a considerable body of research on the role of friendships and parent–child relationships in relation to mental health (10, 29–31), there is very little on the interplay between them. Given that, in this study, there was no association between turning to friends and depressive symptoms but a strong association with turning preferentially to friends, it may be that, at least at this age, it is important to assess the role and quality of friendships in relation to those of parents. An important developmental question then becomes whether this is also true for younger children and whether, later in adolescence, the role of friendships becomes more distinct from that of parents. Similarly, the developmental antecedents of preferentially turning to friends may be different, from turning to parents or to friends. In that case, it will be productive to examine outcomes referring to the child's social system rather than to specific relationship domains. Furthermore, difficulties in accurately identifying the interpersonal resources available in different social domains, not only family and friends but more broadly, may reflect limitations in personality functioning (50, 51). This, in turn, may confer vulnerability to psychopathology in adolescence and adulthood. A prospective examination of the associations we have found in the cross-section is needed before drawing strong conclusions regarding the implications for interventions. If preferentially turning to friends creates vulnerability to depression, particularly in girls, interventions might focus on those scoring highly on this dimension to strengthen the role of parents in responding to the emotional needs of their children in this group.

## Data Availability

Due to ethical constraints supporting data cannot be made openly available. Supporting data are available to bona fide researchers on approval of an application for access. Further information about the data and conditions for access are available at the University of Liverpool Research Data Catalogue: https://doi.org/10.17638/datacat.liverpool.ac.uk/564 and a summary of all data collected is available on https://www.liverpool.ac.uk/population-health/research/groups/first-steps/for-researchers/.

## Footnotes

^1^Assignment to gender and sex is explained in the Methods section
